# Drone inspector for civil infrastructure

**DOI:** 10.1038/s44172-024-00189-1

**Published:** 2024-03-07

**Authors:** Anastasiia Vasylchenkova

**Affiliations:** Communications Engineering, https://www.nature.com/commseng

**Keywords:** Mechanical properties

## Abstract

A recent publication in *Nature Communications* describes the use of cameras on hovering drones to detect submillimetre displacements of bridge spans.

The aging of the civil infrastructure is a challenge for society. Age-related degradation and failure of bridges, tunnels and roads have significant social and economic consequences. To mitigate these risks and ensure undisrupted service, cost-effective and non-destructive monitoring techniques are urgently needed.

Measuring the vertical deflection of bridges caused by wind or traffic load is an essential component of structural integrity assessment. Existing procedures often rely on installable devices, such as accelerometers or laser sensors. But these solutions have associated installation, labour and positioning constraints: there are certain inaccessible places on the bridges where it is not possible to install such sensors. To overcome this limitation, image-based displacement measurement techniques have attracted a lot of interest recently. While showing promising performance, they are also limited by camera positioning constraints, especially when the bridge passes over bodies of water.

Now, a group of researchers from the National Institute of Advanced Industrial Science and Technology in Tsukuba, Japan, have designed an inspection framework and demonstrated its submillimetre displacement accuracy with vision-based methods, at a level appropriate for civil infrastructure monitoring. To achieve this, Shien Ri and colleagues employed a moiré fringe effect. When looking through two overlapping slightly misaligned and partly transparent gratings, for example, two pieces of tulle fabric, or fences on either side of a road, your eye might spot dark areas on the semi-transparent surface. This is the moiré fringe effect. The moiré fringe can be undesirable. For example, it reduces the quality of printing or imaging. However, in metrology, the moiré effect can be helpful. For instance, in one approach known as the sampling moiré method^1^, a high-contrast image created by a slight displacement between overlapping patterns enables a higher accuracy of displacement measurement.

To use the method for bridge monitoring, the researchers used a drone-mounted camera. This trick avoids positioning constraints: the drone can fly almost anywhere. Another important part of the monitoring setup was to position markers at the edges and in the middle of the bridge, see Fig. 1. Measuring the relative displacement of the markers, and using the overlapping phase portraits of the markers with the moiré method, the researchers could measure the slightest difference in the positioning (i.e., displacement), achieving desired submillimetre precision.

**Fig. 1:**
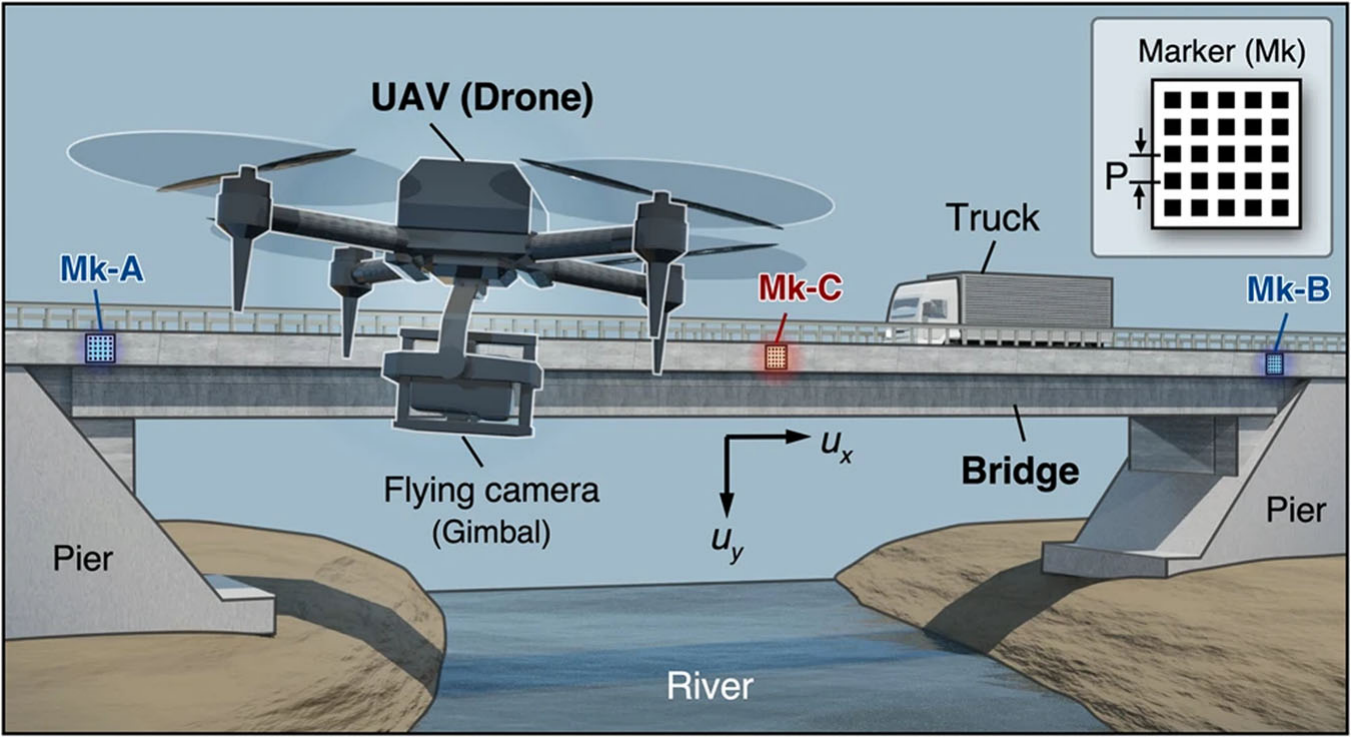
Overview of the measurement system based on drone aerial photography by utilizing the moiré phase analysis methodology. Mk-A, -B and -C are positioning markers, their structure is shown in the inset. UAV is an unmanned aerial vehicle. P is a pitch size. Adapted from *Nature Communications* **15**, 395 (2024).

The choice of drones sets another challenge. As the drone must keep propelling while capturing the footage, it inevitably leads to motion blur affecting image stability. To distinguish between drone movement and bridge movement, the researchers took inspiration from the structure of vestibular sensors. Knowing the actual position of the drone, the researchers employed an active balancing compensation strategy to estimate and compensate for the motion blur.

Ri and colleagues validated the performance of their technique via displacement measurements on a 110 metre concrete bridge spanning a river. They proved that an off-the-shelf drone was sufficient for such a complex task, with all the heavy lifting done by the video processing algorithm. The researchers tested the performance at different distances to the bridge and different span lengths. They acknowledge that the markers’ pitch size limited the resolution, so going beyond 1/100 pixel would not be feasible in their setup. They also expect that heavy weather conditions would reduce the achievable precision.

Nevertheless, with this technique, civil infrastructure monitoring can be performed by an autonomous flying inspector, reducing cost, risks and labour force. Beyond bridge inspection, the researchers highlighted the potential value of this methodology for inspecting heavy industry plants and construction sites, where monitoring would be hazardous for humans to perform.

The original article can be found here: Ri, S., Ye, J., Toyama, N. et al. Drone-based displacement measurement of infrastructures utilizing phase information. *Nat. Commun.* **15**, 395 (2024). 10.1038/s41467-023-44649-2.
